# DNA polymerase λ promotes error-free replication through Watson–Crick impairing N1-methyl-deoxyadenosine adduct in conjunction with DNA polymerase ζ

**DOI:** 10.1016/j.jbc.2021.100868

**Published:** 2021-06-10

**Authors:** Jung-Hoon Yoon, Debashree Basu, Jayati Roy Choudhury, Satya Prakash, Louise Prakash

**Affiliations:** Department of Biochemistry and Molecular Biology, University of Texas Medical Branch, Galveston, Texas, USA

**Keywords:** translesion synthesis, DNA polymerase λ, N1-methyl-deoxyadenosine, error-free TLS by DNA polymerase λ, Hoogsteen base pairing, DNA polymerase ζ, 1-MeA, N1-methyl-deoxyadenosine, (6–4) PPs, (6–4) pyrimidine-pyrimidone photoproducts, CPD, cyclobutane pyrimidine dimer, NC, negative control, nt, nucleotide, Pol, DNA polymerase, TLS, translesion synthesis, W-C, Watson-Crick

## Abstract

In a previous study, we showed that replication through the N1-methyl-deoxyadenosine (1-MeA) adduct in human cells is mediated *via* three different Polι/Polθ, Polη, and Polζ-dependent pathways. Based on biochemical studies with these Pols, in the Polι/Polθ pathway, we inferred a role for Polι in the insertion of a nucleotide (nt) opposite 1-MeA and of Polθ in extension of synthesis from the inserted nt; in the Polη pathway, we inferred that this Pol alone would replicate through 1-MeA; in the Polζ pathway, however, the Pol required for inserting an nt opposite 1-MeA had remained unidentified. In this study, we provide biochemical and genetic evidence for a role for Polλ in inserting the correct nt T opposite 1-MeA, from which Polζ would extend synthesis. The high proficiency of purified Polλ for inserting a T opposite 1-MeA implicates a role for Polλ—which normally uses W-C base pairing for DNA synthesis—in accommodating 1-MeA in a *syn* confirmation and forming a Hoogsteen base pair with T. The potential of Polλ to replicate through DNA lesions by Hoogsteen base pairing adds another novel aspect to Polλ’s role in translesion synthesis in addition to its role as a scaffolding component of Polζ. We discuss how the action mechanisms of Polλ and Polζ could be restrained to inserting a T opposite 1-MeA and extending synthesis thereafter, respectively.

Translesion synthesis (TLS) DNA polymerases (Pols) exhibit a high specificity for replicating through different types of DNA lesions. Whereas replication through certain DNA lesions can be performed by just one Pol, such as by Polη opposite cyclobutane pyrimidine dimers (CPDs) (1, 2, 3, 4, 5, 6), replication through a vast array of DNA lesions requires the sequential action of two Pols, wherein one Pol inserts a nucleotide (nt) opposite the DNA lesion and another Pol extends synthesis from the inserted nt. Biochemical and structural studies with yeast Polζ have provided strong evidence for its role in extending synthesis from nts inserted opposite DNA lesions by other TLS Pols (7, 8, 9, 10), and genetic evidence accrued from TLS studies opposite a number of DNA lesions in human cells aligns with such a Polζ role (5, 6, 11, 12).

In yeast or cancer cells, Rev1 functions as a scaffolding component of Polζ and TLS by Rev1-Polζ operates in a highly error-prone manner (13, 14, 15, 16, 17, 18). In normal human cells, however, Rev1 functions as an indispensable scaffolding component of the Y-family Pols η, ι, and κ; and TLS studies opposite a number of DNA lesions have indicated that Rev1-dependent TLS by Y-family Pols operates in a much more error-free manner in human cells than indicated from the fidelity of the purified pols (11, 19, 20, 21). Furthermore, in a recent study we provided evidence for an indispensable role of Polλ as a scaffolding component of Polζ; and from TLS studies opposite a number of DNA lesions, we inferred that Polλ-dependent TLS by Polζ operates in a predominantly error-free manner in human cells (22). In that study we analyzed Polλ’s role in TLS opposite the UV lesions CPDs and (6–4) pyrimidine-pyrimidone photoproducts (6–4) PPs, the oxidative DNA lesion thymine glycol (Tg), and the 1,N^6^-ethenodeoxyadenosine (εdA) lesion—formed in DNA through interaction with aldehydes derived from lipid peroxidation. In TLS opposite CPD, Tg, and εdA, Polζ extends synthesis from the nt inserted opposite the lesion site by another DNA Pol; and although Polλ is indispensable for Polζ’s role in TLS opposite these DNA lesions, its DNA polymerase activity is not required. Thus, for TLS opposite these DNA lesions, only Polλ’s scaffolding activity is required (22). For TLS opposite (6–4) PPs, however, Polλ’s polymerase activity is also required, and Polλ promotes error-free replication through this lesion in human and mouse cells (22). Since (6–4) TT PP induces a large structural distortion in DNA and since it impairs the ability of the 3’T to form a normal Watson–Crick (W-C) base pair with the correct nt (23, 24, 25, 26), it remains unclear how Polλ, which uses W-C base pairing for normal DNA synthesis, manages error-free TLS opposite this DNA lesion.

N1-methyl-deoxyadenosine (1-MeA) is repaired by direct demethylation, primarily by the ABH2 enzyme in human cells (27). The evidence that 1-MeA residues accumulate over time in the genomic DNA of the livers from ABH2 null mice has indicated that endogenous DNA methylation contributes to their formation (27). In human cells, TLS through the 1-MeA adduct is mediated *via* three independent pathways in which Polι and Polθ function in one pathway and Pols η and ζ function in the other two pathways, respectively (28). TLS by all three pathways operates in a predominantly error-free manner in human cells. For the Polι/Polθ pathway, following nt insertion by Polι opposite 1-MeA by forming a Hoogsteen base pair with the T residue (29), Polθ would extend synthesis, whereas in the Polη pathway, Polη would perform both the steps of TLS (28). Our evidence for the requirement of Polλ as an indispensable scaffolding component of Polζ strongly suggested that it would be required for Polζ-dependent TLS opposite 1-MeA; further, it raised the possibility that Polλ may insert the correct nt opposite 1-MeA from which Polζ could extend synthesis.

Here we provide genetic and biochemical evidence for the role of Polλ in conjunction with Polζ in mediating error-free replication through 1-MeA by inserting the correct nt opposite it. We discuss how by adopting Hoogsteen base pairing as a mechanism for inserting the correct nt opposite 1-MeA, Polλ could promote error-free replication through this adduct.

## Results

### Requirement of Polλ for TLS opposite 1-MeA in conjunction with Polζ

In our previous analyses of the genetic control of TLS opposite 1-MeA in human cells, we identified the involvement of three independent Polι/Polθ, Polη, and Polζ pathways (28). In the Polζ pathway, however, the identity of the Pol that could insert an nt opposite 1-MeA had remained unknown. To determine whether Polλ functions together with Polζ, we analyzed the effects of siRNA depletion of Polλ alone and in combination with depletion of other TLS Pols on TLS frequency opposite 1-MeA carried on the leading strand template in the duplex plasmid in which bidirectional replication initiates from an origin of replication (28).

As shown in Table 1, TLS in normal human fibroblasts (HFs) treated with control (NC) siRNA occurs with a frequency of ∼63%. In Polη depleted cells, TLS frequency is reduced to ∼53% and depletion of Polι, Polθ, Rev3, or Polλ reduced TLS frequency to 40 to 46%. Our evidence that codepletion of Polλ with Polη, Polι, or Polθ reduces TLS frequency nearly to ∼27% indicated a role for Polλ in a TLS pathway independent of Polη or Polι/Polθ pathways, and our observation that TLS frequency remains the same in cells codepleted for Polλ and Rev3 (∼42%) as in cells depleted for either Pol alone implicated a role for Polλ in TLS in conjunction with Polζ.

**Table 1:** Effects of siRNA knockdowns of Polλ and other TLS Pols on replicative bypass of 1-MeA carried on the leading DNA strand template in normal human fibroblasts

siRNA	Number of *Kan*^*+*^ colonies	Number of blue colonies among *Kan*^*+*^ colonies	TLS (%)
NC	407	258	63.4
Polη	368	196	53.3
Polι	356	165	46.3
Polθ	475	189	39.8
Rev3	344	157	45.6
Polλ	436	204	46.8
Polη + Polλ	390	104	26.7
Polι + Polλ	408	114	27.9
Polθ + Polλ	417	112	26.9
Rev3 + Polλ	317	134	42.3

**Figure 1:**
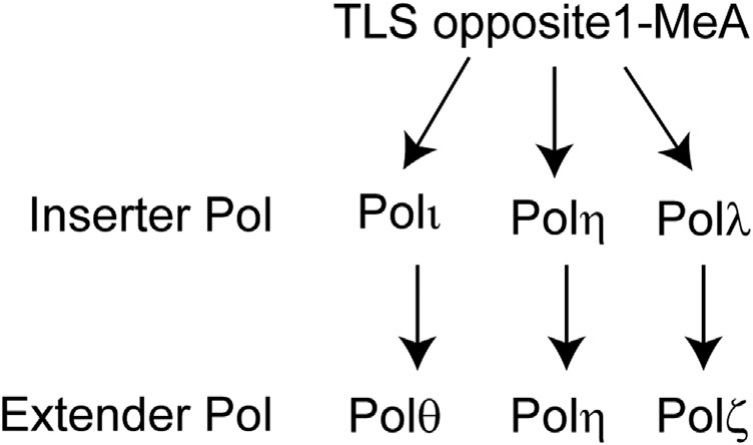
**TLS pathways for replication through 1-MeA.** In the Polι/Polθ pathway, following nt insertion by Polι by Hoogsteen base pairing opposite 1-MeA, Polθ would extend synthesis; in the Polη pathway, this Pol would act alone at both the steps of TLS. TLS by Polι and Polη requires the noncatalytic and scaffolding role of Rev1. In the Polλ/Polζ pathway, following the insertion of T opposite 1-MeA by Polλ, Polζ would extend synthesis. In this pathway, Polλ’s scaffolding role would be additionally required for assembly with Polζ.

To provide further evidence for the role of Polλ in TLS with Polζ, we analyzed the effects or Polλ depletion alone and in combination with the depletion of other TLS Pols in XPV HFs (Table 2). In control siRNA-treated XPV HFs, TLS opposite 1-MeA occurs with a frequency of ∼47% and as expected from the role of Polι/Polθ and Polλ/Polζ in Polη-independent pathways, TLS frequency is reduced to ∼30% in XPV HFs depleted for Polι, Polθ, Rev3, or Polλ (Table 2). Our results that TLS frequency is reduced to ∼5% in XPV HFs codepleted for Polλ either with Polι or with Polθ and that TLS frequency remains nearly the same (∼29%) in XPV HFs codepleted for Polλ and Rev3 as in cells depleted for either Pol alone add further support for the role of Polλ in TLS opposite 1-MeA together with Polζ and independent of Polι and Polθ. Altogether from TLS analyses in WT HFs and XPV HFs, we conclude that TLS through 1-MeA operates *via* three independent Polι/Polθ, Polη, and Polλ/Polζ pathways (Fig. 1).

**Table 2:** Effects of siRNA knockdowns of Polλ and other TLS Pols on replicative bypass of 1-MeA carried on the leading DNA strand template in XPV human fibroblasts

siRNA	Number of *Kan*^*+*^ colonies	Number of blue colonies among *Kan*^*+*^ colonies	TLS (%)
NC	396	180	45.5
Polι	410	124	30.2
Polθ	395	110	27.8
Rev3	426	129	30.3
Polλ	502	145	28.9
Polι + Polλ	230	11	4.8
Polθ + Polλ	426	23	5.4
Rev3 + Polλ	472	136	28.8

### Requirement of Polλ’s polymerase activity for TLS opposite 1-MeA

To determine if Polλ’s polymerase activity was required for TLS opposite 1-MeA, we analyzed the effects of the D427A, D429A mutations, which inactivate this activity. For these studies, we stably expressed siRNA-resistant wild-type human Polλ or the D427A, D429A catalytic mutant Polλ in WT HFs. As shown in Table 3, TLS opposite 1-MeA in Polλ-depleted HFs harboring the vector plasmid occurs with a frequency of ∼45%, and the frequency rises to ∼64% in cells expressing WT Polλ. Our results that TLS frequency is reduced to the same level (∼45%) in cells expressing the D427A, D429A catalytic mutant as in cells harboring the vector plasmid establish the requirement of Polλ’s polymerase activity for TLS through 1-MeA in human cells. Additionally, we confirmed the requirement of Polλ’s polymerase activity for TLS through this adduct in Polλ^−/−^ MEFs (Table 4).

**Table 3:** Effects of catalytically active (WT) Polλ, catalytically inactive D427A, D429A Polλ, N-terminally deleted (245–575) Polλ, catalytically active (WT) Rev1, catalytically inactive D570A, E571A Rev1, catalytically active (WT) Rev3, or catalytically inactive D2781A, D2783A Rev3 on TLS opposite 1-MeA carried on the leading DNA strand template in normal human fibroblasts

Vector expressing	siRNA	Number of *Kan*^*+*^ colonies	Number of blue colonies among *Kan*^*+*^ colonies	TLS (%)
No Polλ (control)	Polλ	372	166	44.6
WT (1–575) Polλ	Polλ	326	208	63.8
D427A, D429A Polλ	Polλ	306	138	45.1
(245–575) Polλ	Polλ	334	216	64.7
No Rev3 (control)	Rev3	294	124	42.2
WT Rev3	Rev3	242	161	66.5
D2781A, D2783A Rev3	Rev3	409	180	44.0
No Rev1 (control)	Rev1	354	106	29.9
WT Rev1	Rev1	484	302	62.4
D570A, E571A Rev1	Rev1	248	158	63.7

**Table 4:** Effects of catalytically active (WT) Polλ, catalytically inactive D427A, D429A Polλ, or N-terminally deleted (245–575) Polλ on TLS opposite 1-MeA carried on the leading DNA strand template in Polλ−/− MEFs

Vector expressing	Number of *Kan*^*+*^ colonies	Number of blue colonies among *Kan*^*+*^ colonies	TLS (%)
No Polλ (control)	340	146	42.9
WT Polλ	318	194	61.0
D427A, D429A Polλ	375	153	40.8
(245–575) Polλ	276	170	61.6

### Polλ’s BRCT domain is not required for TLS opposite 1-MeA

Polλ is a 575 residue polypeptide that contains an N-terminal BRCT domain. We have shown previously that N-terminally deleted Polλ comprised of residues 245 to 575, which lacks the BRCT domain and the proline-rich region, physically interacts with the Rev7 subunit of Polζ and that this N-terminally deleted Polλ supports TLS through (6–4) TT photoproduct in human cells (22). Our results that expression of (245–575) Polλ in HFs supports WT levels of TLS (Table 3) confirm that the N-terminal BRCT domain and the adjoining proline-rich region are also not required for Polλ’s role in TLS through 1-MeA in HFs (Table 3); additionally, we confirmed these results in Polλ^−/−^ MEFs (Table 4).

### Purified Polλ conducts error-free TLS through 1-MeA

**Figure 2:**
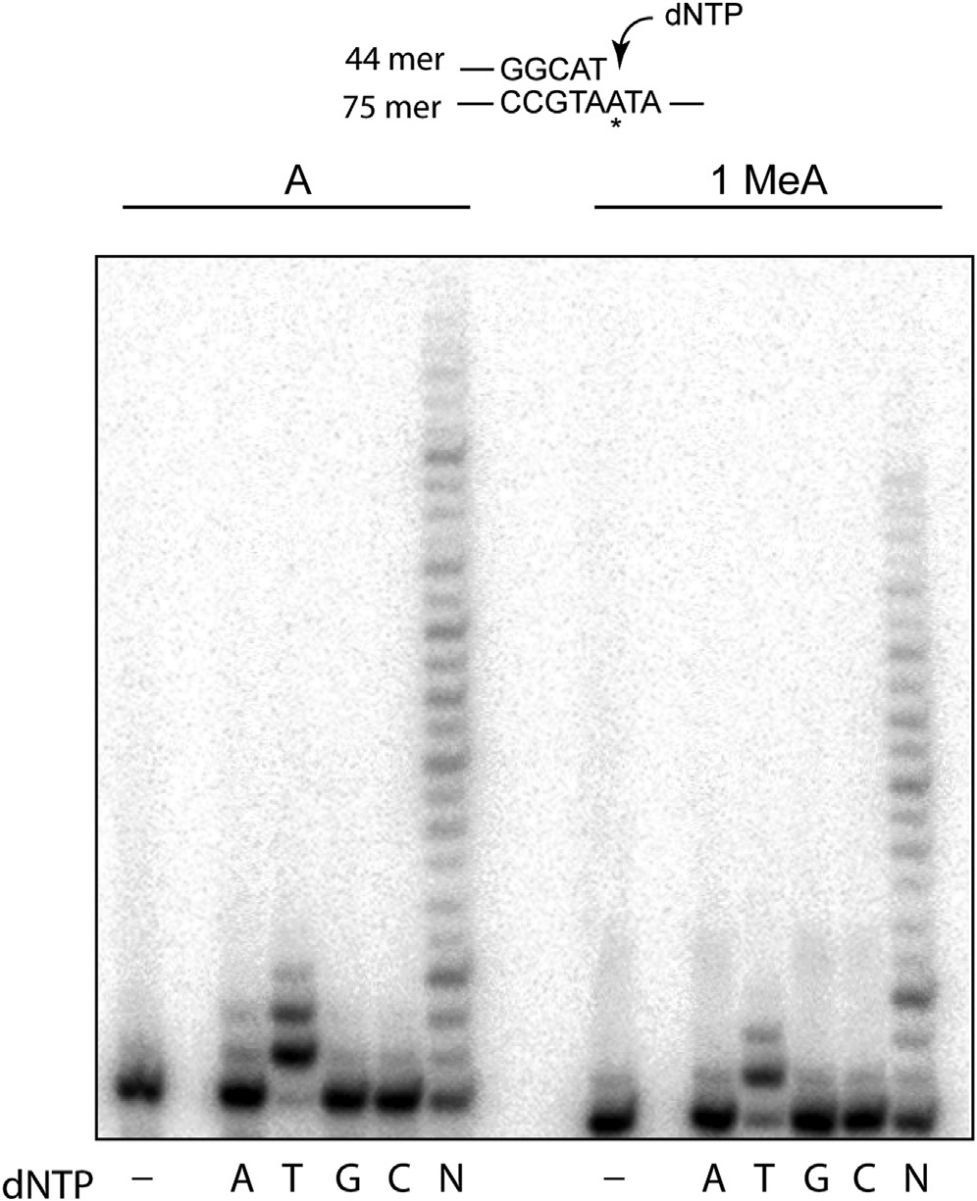
**Deoxynucleotide incorporation by Polλ opposite undamaged A and 1-MeA template.** Polλ (10 nM) was incubated with template DNA (5 nM) and with 100 μM of a single nucleotide (A, T, G, or C) or 100 μM of each of the four dNTPs (N) for 15 min at 37 °C. A schematic representation of the DNA template and primer used is shown above the gel; the *asterisk* indicates the site of an A or 1-MeA in the template DNA.

The requirement of Polλ’s polymerase activity for TLS through the 1-MeA adduct in conjunction with Polζ in human cells suggested that Polλ would insert an nt opposite 1-MeA from which Polζ would extend synthesis. Hence, we examined purified Polλ for its ability to insert dATP, dTTP, dGTP, or dCTP opposite 1-MeA and to synthesize DNA through the adduct in the presence of all four dNTPs. As shown in Figure 2, Polλ replicates through the undamaged template residue A by inserting a T. Opposite 1-MeA also Polλ inserts a T and then extends synthesis similar to that on undamaged DNA.

The high proficiency of Polλ for inserting the correct nt opposite 1-MeA stands in sharp contrast to the error-proneness of purified Polι or Polη opposite this adduct (28). Thus in addition to the insertion of a T, Polι inserts an A or a C opposite 1-MeA, and steady-state kinetic analyses have indicated that it does so with only an ∼100-fold lower catalytic efficiency than for the insertion of correct T (29). Likewise, compared with the catalytic efficiency for the insertion of a T, Polη inserts an A or a G opposite 1-MeA with only an ∼40-fold reduction in catalytic efficiency, and it inserts a C opposite 1-MeA with only an ∼100-fold reduction in catalytic efficiency (Table 5). Nevertheless, in spite of their error-proneness *in vitro*, both these Pols conduct predominantly error-free TLS through 1-MeA in human cells (28).

**Table 5:** Steady-state kinetic analyses of nucleotide incorporation opposite undamaged A or 1-MeA by human Polη

Template residue	Incoming nucleotide	*k*_*cat*_ (min^−1^)	*K*_*m*_ (μM)	*k*_*cat*_/*K*_*m*_	Catalytic efficiency relative to T
A	T	7.4 ± 0.2	0.39 ± 0.08	19	1
	A	2.2 ± 0.1	2.4 ± 0.4	0.9	5 × 10^−2^
	G	2.9 ± 0.2	5.3 ± 1.3	0.54	3 × 10^−2^
	C	2.2 ± 0.18	13.46 ± 2	0.16	0.8 × 10^−2^
1-MeA	T	5.8 ± 0.3	1.05 ± 0.2	5.8	1
	A	2.3 ± 0.5	14.99 ± 6	0.15	2.6 × 10^−2^
	G	1.6 ± 0.2	10.58 ± 3.7	0.15	2.6 × 10^−2^
	C	1.9 ± 0.09	35.9 ± 5.3	0.05	1 × 10^−2^

Polη (0.25 nM) was incubated with primer:template DNA substrate (10 nM) and increasing concentrations of dNTPs for 10 min, at 37 °C. The nucleotide incorporation rate was plotted against dNTP concentration and the data were fit to the Michaelis–Menten equation. Apparent K_m_ and k_cat_ values were obtained from the fit and used to calculate the efficiency of deoxynucleotide incorporation (k_cat_/K_m_).

### Requirement of Polζ polymerase activity for TLS through the 1-MeA adduct in human cells

The ability of purified Polλ for replicating through the 1-MeA adduct with nearly the same proficiency as for replicating undamaged DNA raised the question of whether Polζ’s polymerase activity was required for TLS through this adduct in human cells. To examine this, we expressed full-length WT Rev3, or the D2781A, D2783A mutant Rev3, defective in its polymerase activity, in HFs. In Rev3-depleted HFs harboring the vector plasmid, TLS opposite 1-MeA occurs with a frequency of ∼42% and TLS frequency rises to ∼66% in cells expressing WT Rev3. Our results that in cells expressing the D2781A, D2783A Rev3 catalytic mutant, TLS is reduced to the same level (∼44%) as in cells harboring the vector control (Table 3) confirm that the Rev3 DNA polymerase activity is, in fact, required for TLS through 1-MeA in human cells.

### Requirement of noncatalytic role of Rev1 for TLS opposite 1-MeA in conjunction with Polι and Polη

In previous studies opposite a number of DNA lesions, we have provided evidence for a scaffolding role of Rev1 in TLS by Y-family Pols (11, 19, 20, 21). To confirm that Rev1 plays a similar role in TLS by Polι and Polη opposite 1-MeA, we analyzed the epistatic relationship of Rev1 with these Pols. Our results that TLS occurs at the same frequency in Rev1 depleted HFs (∼30%) as in cells depleted for Rev1 together with Polη or with Polι and that TLS frequency is reduced to ∼6% in cells depleted for Rev1 together with Rev3 or with Polλ (Table 6) concur with a role of Rev1 in TLS in Polι and Polη-dependent pathways that operate independently or Polλ/Polζ pathway. To confirm that Rev1’s polymerase activity is not required for TLS opposite 1-MeA, we expressed full-length WT Rev1, or D570A, E571A Rev1 defective in its DNA polymerase activity, in HFs. Our results that the catalytic mutant Rev1 supports the same level of TLS (∼64%) as the WT Rev1 (∼62%) (Table 3) confirmed that Rev1’s polymerase activity was not required. Thus, only the scaffolding role of Rev1 is required for the Polι and Polη TLS pathways.

**Table 6:** Effects of siRNA knockdowns of Rev1 and other TLS Pols on replicative bypass of 1-MeA carried on the leading DNA strand template in normal human fibroblasts

siRNA	Number of *Kan*^*+*^ colonies	Number of blue colonies among *Kan*^*+*^ colonies	TLS (%)
NC	476	307	64.5
Rev1	340	104	30.6
Polη + Rev1	378	112	29.6
Polι + Rev1	402	120	29.9
Rev3 + Rev1	382	22	5.8
Polλ + Rev1	420	24	5.7

## Discussion

### Hoogsteen base pairing as a mechanism for nt insertion opposite 1-MeA by Polλ

Since the addition of a methyl group to the N1 atom of deoxyadenosine disrupts W-C base pairing (29), the insertion of T opposite 1-MeA by Polλ could occur only if the adduct is accommodated in a *syn* conformation in its active site and the adduct forms a Hoogsteen base pair with T. Thus Polλ active site, which normally accommodates template residues in an *anti* conformation and forms a W-C base pair with the incoming nt (30), would stabilize 1-MeA in a *syn* conformation. Such an ability of Polλ active site to accommodate a W-C impairing DNA lesion in a *syn* conformation would add another novel aspect to Polλ’s function in TLS—in addition to its role as a scaffolding component of Polζ for TLS in human cells.

### Modulation of the action mechanism of Polλ and Polζ for TLS through 1-MeA

Even though purified Polλ inserts a T opposite 1-MeA and extends synthesis, Polζ’s polymerase activity is still required for replication through 1-MeA in human cells. The requirement of both the Polλ and Polζ polymerase activities strongly suggests that their polymerase activities are restrained to act at the nt insertion or the extension step of TLS in human cells. We presume that in the multiprotein ensemble of Polλ-Polζ, the action mechanism of the two Pols is restrained such that Polλ’s action is limited to inserting a T opposite 1-MeA and Polζ functions at the extension step. The decipherment of the action mechanism of these Pols in human cells would require the identification of the components of the Polλ-Polζ multiprotein ensemble and biochemical analyses of Polλ’s and Polζ’s role in TLS opposite 1-MeA in the Polλ-Polζ ensemble.

### Role of Rev1 in the formation of multiprotein ensembles of Y-family Pols

Our evidence that similar to the requirement of Rev1 as a scaffolding component of Y-family Pols for TLS opposite CPDs, (6–4) PPs, 3-methyl deoxyadenosine, εdA, and other DNA lesions, Rev1’s scaffolding role is required for TLS opposite 1-MeA by Polι and Polη suggests that Rev1 would effect the assembly of these Y-family Pols with the other protein components and that the fidelity of Polι and Polη for TLS opposite 1-MeA would be elevated in the respective multiprotein ensemble thus formed.

### Hoogsteen base pairing by Polι for TLS opposite 1-MeA and modulation of its fidelity in human cells

Polι differs from other Y-family Pols in that its active site accommodates a template purine A or G in a *syn* conformation, which then forms a Hoogsteen base pair with the incoming nt (31, 32, 33). This allows Polι to accommodate DNA lesions, such as εdA, which impair W-C base pairing, in a *syn* conformation and to form a Hoogsteen base pair with the incoming nt (9). However, biochemical studies with Polι have indicated that it can insert not only the correct nt T opposite εdA but also the incorrect nt C with only a few-fold reduction in catalytic efficiency, and structure studies have shown that εdA in Polι active site adopts a *syn* conformation and that it Hoogsteen base pairs with the incoming dTTP or dCTP (9). Nevertheless, in spite of the penchant of Polι for inserting a C opposite εdA, Polι conducts error-free TLS opposite this adduct in human cells (11). Similar to εdA, 1-MeA is accommodated in a *syn* conformation in Polι’s active site, and it forms a Hoogsteen base pair with T (29); however, even though purified Polι misincorporates an A or a C at a frequency of ∼10^−2^ (29), it promotes error-free TLS through 1-MeA in human cells. The adoption of entirely error-free TLS opposite εdA and of predominantly error-free TLS opposite 1-MeA in human cells could be explained if Polι’s error-proneness is annulled in the multiprotein ensemble that the scaffolding role of Rev1 would assemble.

### Hoogsteen base pairing by Polη for nt insertion opposite 1-MeA

Polη uses W-C base pairing for replicating undamaged DNA (34) and for replicating through the two covalently linked pyrimidines of a CPD (2, 4, 35, 36, 37). And Polη can replicate through both the guanines of a cisplatin GG intrastrand cross-link with both the Gs in the cross-link forming a W-C base pair with the incoming dCTP (38, 39). Hence, the hallmark of Polη has been its ability to accommodate two template residues in its active site and to form a W-C base pair with the incoming nt. The proficiency of purified Polη for incorporating a T opposite 1-MeA and the genetic evidence that Polη replicates through this adduct in human cells strongly suggest that Polη accommodates 1-MeA in its active site in a *syn* conformation, which then Hoogsteen base pairs with the correct nt T or with incorrect nts. We presume that the intrinsic error-proneness of Polη for TLS through 1-MeA is attenuated in the Polη multiprotein ensemble.

## Experimental procedures

### Construction of plasmid vectors containing 1-MeA

The heteroduplex vectors containing 1-MeA on the leading or the lagging strand template were constructed as described previously (28).

### Cell lines and cell culture

Normal human fibroblasts (Coriell Institute Cell Repository, GM00637), XPV fibroblasts (Coriell Institute Cell Repository, GM03617), Polλ^−/−^ MEFs, and big blue mouse embryonic fibroblasts (Agilent) were grown in DMEM medium (GenDEPOT) containing 10% fetal bovine serum (GenDEPOT) and 1% antibiotic-antimycotic (GenDEPOT). Cells were grown on plastic culture dishes at 37 °C in a humidified incubator with 5% CO_2_.

### Translesion synthesis assays in HFs and Polλ^−/−^ MEFs

The siRNA sequences, the siRNA knockdown efficiency of TLS Pols, as well as the detailed methods for TLS assays and for mutational analyses have been described previously (6, 11, 22, 40).

### Stable expression of wild-type and catalytic mutant Rev1, Rev3, and Polλ, and N-terminally deleted Polλ

Stable expression of siRNA resistant WT or D570A E571A mutant Rev1 has been described previously (19). Stable expressions of siRNA-resistant WT Polλ, catalytic mutant (D427A D429A) Polλ, N-terminally deleted (245–575) Polλ, and of siRNA-resistant WT Rev3 or catalytic mutant (D2781A D2783A) were done as described (22).

### DNA polymerase assays with Polλ

The template 75-mer oligonucleotide contained the sequence 5′ AGC AAG TCA CCA ATG TCT AAG AGT TCG TAT AAT GCC TAC ACT GGA GTA CCG GAG CAT CGT CGT GAC TGG GAA AAC-3′, and it harbored an undamaged A or a 1-MeA at the underlined position. For examining the incorporation of dATP, dTTP, dCTP, or dGTP nucleotides individually, or of all four dNTPs, a 44 mer primer 5′ GTT TTC CCA GTC ACG ACG ATG CTC CGG TAC TCC AGT GTA GGC AT-3′ was annealed to the abovementioned 75 mer template. The specific details of DNA synthesis assays are stated in Figure 2 legend, and the general methods for DNA synthesis assays were as described before (22, 28).

### Steady-state kinetic analyses

Steady-state kinetic analyses for deoxynucleotide incorporation opposite undamaged A or 1-MeA by Polη were performed as described (21, 41). The specific details for kinetic analyses are described in Table 5 legend.

## Data availability

All relevant data are contained within the article.

## Conflict of interest

The authors declare that they have no conflicts of interest with the contents of this article.
